# Supplementary figures and images for: Interplay between de novo and salvage pathways of GDP-fucose synthesis
Source: PLoS One. 2024 Oct 24;19(10):e0309450. doi: 10.1371/journal.pone.0309450 (PMC11501016; doi:10.1371/journal.pone.0309450)

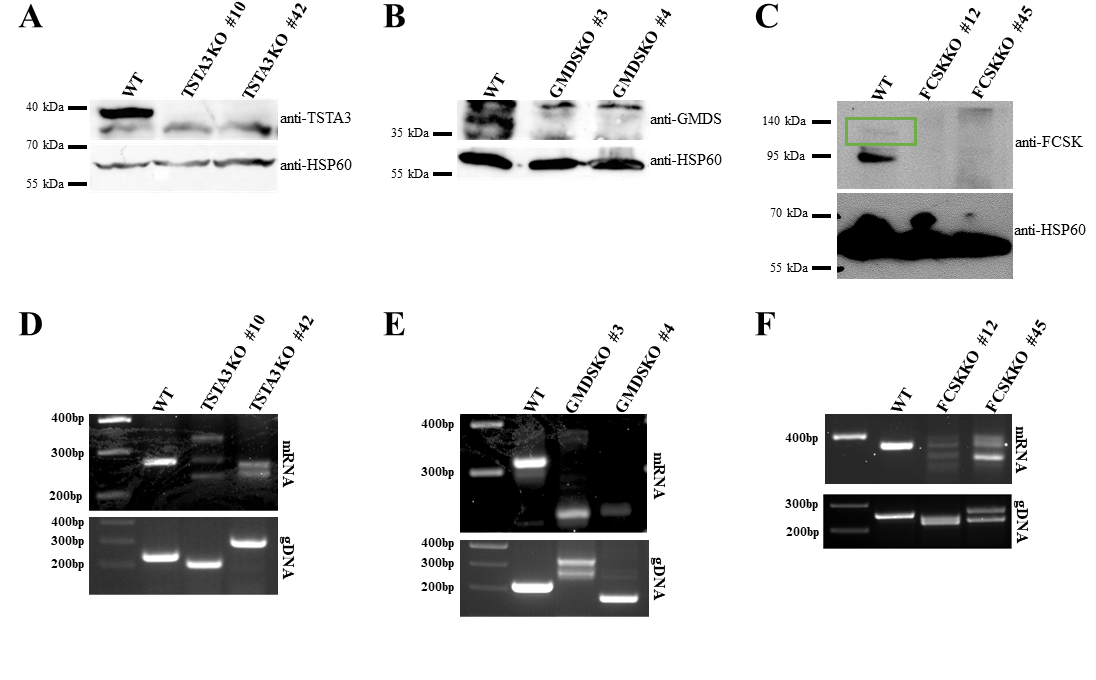

Supplement: S1 Fig — (A) TSTA3 western blotting analysis in wild-type and TSTA3KO HEK293T cell lines. Anti-HSP60B antibody was used as a loading control. (B) GMDS western blotting analysis in wild-type and GMDSKO HEK293T cell lines. An anti-HSP60 antibody was used as a loading control. (C) FCSK western blotting analysis in wild-type and FCSKKO HEK293T cell lines. A green frame indicates an appropriate band coming from FCSK in wild-type cells. The anti-HSP60 antibody was used as a loading control. (D) Verification of a knockout of the TSTA3 gene in HEK293T cell line. Total RNA and genomic DNA (gDNA) were isolated from the wild-type (WT) TSTA3 knockout (TSTA3KO) cells, and either PCR (DNA) or RT-PCR (mRNA) was performed using TSTA3 gene-specific primers. (E) Verification of a knock-out of the GMDS gene in HEK293T cell line. Total RNA and genomic DNA (gDNA) were isolated from the wild-type (WT) and GMDS knockout (GMDSKO) cells, and either PCR (DNA) or RT-PCR (mRNA) was performed using GMDS gene-specific primers. (F) Verification of a knockout of the TSTA3 gene in HEK293T cell line. Total RNA and genomic DNA (gDNA) were isolated from the wild-type (WT) TSTA3 knockout (TSTA3KO) cells, and either PCR (DNA) or RT-PCR (mRNA) was performed using TSTA3 gene-specific primers. (G) Verification of a knock-out of the FCSK gene in HEK293T cell line. Total RNA and genomic DNA (gDNA) were isolated from the wild-type (WT) and FCSK knockout (GMDSKO) cells, and either PCR (DNA) or RT-PCR (mRNA) was performed using FCSK gene-specific primers. (TIF) [file pone.0309450.s001.tif]

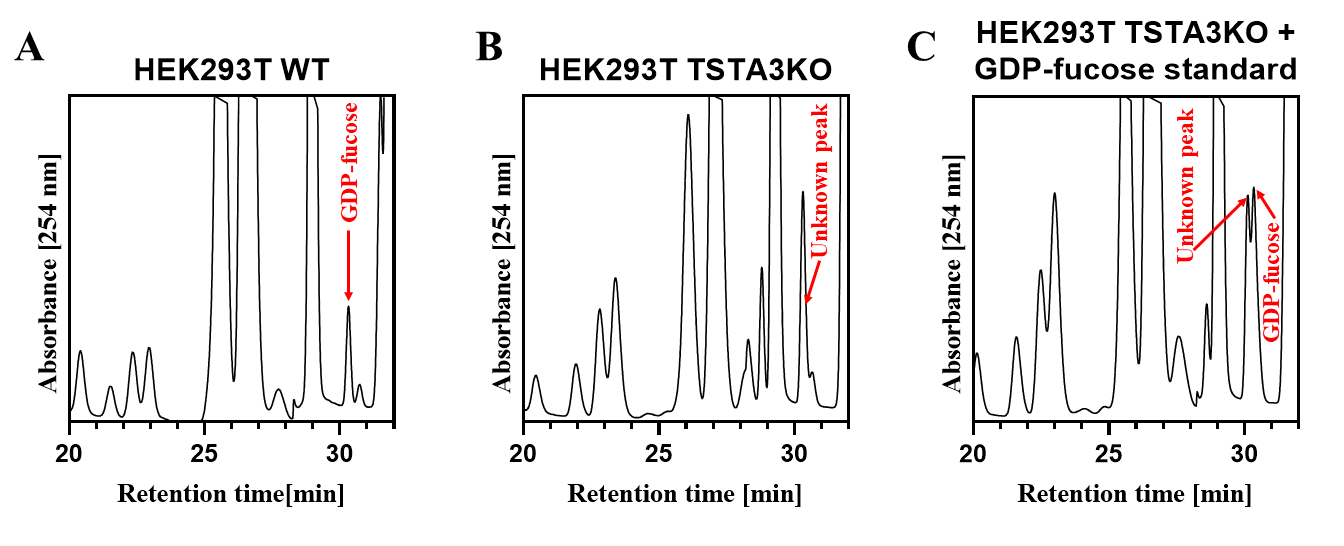

Supplement: S2 Fig — Cell lysates of (A) HEK293T WT, (B) HEK293T TSTA3KO, (C) HEK293T TSTA3KO with the addition of GDP-fucose standard were subjected to nucleotide sugar extraction, and then RP-HPLC separation. Peaks correspond to GDP-fucose, and unknown ones in TSTA3KO cells are signed and indicated by arrows. (TIF) [file pone.0309450.s002.tif]

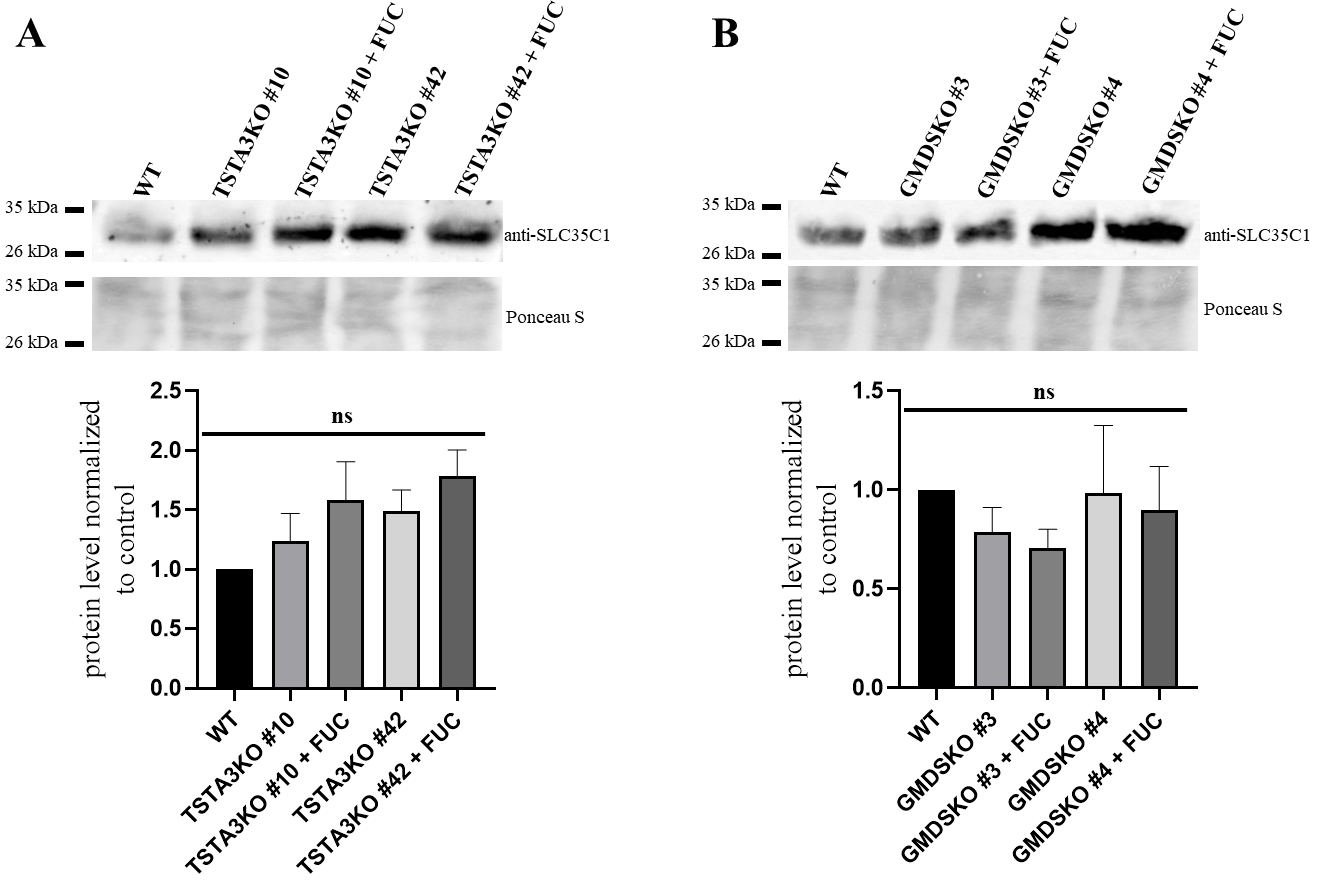

Supplement: S3 Fig — Cell lysates were applied for western blotting of SLC35C1 protein in (A) TSTA3KO and (B) GMDSKO cell lines either fed with fucose or not and compared to wild-type cells. Ponceau S staining was used as a loading control for all western blotting experiments. Data are represented as mean ± SEM. Each sample was run at least in three biological replicates. ns, not significant, as determined using one-way ANOVA with the Tukey post-hoc test. (TIF) [file pone.0309450.s003.tif]
