# Supplementary material for: Interplay between de novo and salvage pathways of GDP-fucose synthesis
Source: PLoS One. 2024 Oct 24;19(10):e0309450. doi: 10.1371/journal.pone.0309450 (PMC11501016; doi:10.1371/journal.pone.0309450)
Supplement: S1 File — (ZIP) [file pone.0309450.s004.zip › Supporting Informations_Tables.docx]

**S1 Table. List of primers used for verification of genes inactivation.**

| **No.** | **Primer name** | **Primer sequence 5ʹ-3ʹ** | **Product length [bp]** |
| --- | --- | --- | --- |
| *Primers used in RT-PCR analysis with total RNA as a template* | | | |
| 1 | TSTA3KO_FP | TTTGTCTCCTCTAAAGACGC | 268 |
| 2 | TSTA3KO_RP | TCTCATCTATCGGGTAGGTC | 268 |
| 3 | GMDSKO_FP | AGATGGGCAAGCCCAGGAAC | 220 |
| 4 | GMDSKO_RP | GTACTGTCAGTGAGATCGCCA | 220 |
| 5 | FCSKKO_FP | CACATGGGTCGAGACTTC | 331 |
| 6 | FCSKKO_RP | CCTGGTAGTAAATGTCCAAAA | 331 |
| *Primers used in PCR analysis with genomic DNA as a template* | | | |
| 7 | TSTA3KO_FP | ACCCTCCCTTTTGACTGT | 232 |
| 8 | TSTA3KO_RP | TACCCAGAAGTCCAAATTGT | 232 |
| 9 | GMDSKO_FP | CAGGTCCATGGAATTGTACG | 313 |
| 10 | GMDSKO_RP | ATAGCCTTCTAACAGCCAAA | 313 |
| 11 | FCSKKO_FP | CTTCCAGGGTCGAGACTT | 260 |
| 12 | FCSKKO_RP | TAACATTAGGCACCCTCAAA | 260 |

**S2 Table. List of primers used in the generation of FCSK and FPGT overexpressing constructs.**

| **No.** | **Primer name** | **Primer sequence 5ʹ-3ʹ** |
| --- | --- | --- |
| 1 | HA_FCSK_FP | AATGTCGACATGGCATACCCATACGACGTACCAGACTACGC  AATGGAGCAGCCGAAGGGAG |
| 2 | HA_FCSK_RP | ATTGCTAGCTCATGGGAAAGGGCAACAGG |
| 3 | cmyc_FPGT_FP | AATGTCGACATGGCAGAACAAAAACTTATTTCTGAAGAAGA  TCTGATGCGTGCTGTGCGGCGCG |
| 4 | cmyc_FPGT_RP | AATAGCGCTCTACATCAAACTGCTTTTTAAAC |

**S3 Table. List of antibodies used in western blotting analysis.**

| **Antibody** | **Origin** | **Dilution** | **Manufacturer** |
| --- | --- | --- | --- |
| anti-FCSK | rabbit | 1:50 | Novus Biologicals |
| anti-FPGT | rabbit | 1:100 | Novus Biologicals |
| anti-GMDS | rabbit | 1:100 | Novus Biologicals |
| anti-TSTA3 | rabbit | 1:100 | Novus Biologicals |
| anti-FUCA1 | rabbit | 1:100 | Proteintech |
| anti-FUOM | rabbit | 1:50 | Cusabio |
| anti-CaSR | mouse | 1:50 | Santa Cruz Biotechnology |
| anti-calnexin | mouse | 1:1000 | Proteintech |
| anti-calnexin | rabbit | 1:1000 | Abcam |
| anti-GLUT1 | rabbit | 1:1000 | Proteintech |
| anti-HSP60 | mouse | 1:50000 | Santa Cruz Biotechnology |
| anti-GAPDH | mouse | 1:10000 | Abcam |
| anti-HA | rabbit | 1:1000 | Proteintech |
| anti-SLC35C1 | rabbit | 1:1000 | Thermo Fisher Scientific |
| anti-mouse HRP | goat | 1:10000 | Promega |
| anti-rabbit HRP | goat | 1:10000 | Sigma-Aldrich |
