# Supplementary material for: Interplay between de novo and salvage pathways of GDP-fucose synthesis
Source: PLoS One. 2024 Oct 24;19(10):e0309450. doi: 10.1371/journal.pone.0309450 (PMC11501016; doi:10.1371/journal.pone.0309450)

Page 12.

- Used as an insert in figure 4A: western blotting of FPGT

The MW marker is marked on the left side of the line (70 kDa and 55 kDa). The order of samples from left to right: line 1-HEK293T WT (wild type), line 2-GMDSKO #3, line 3 -GMDSKO #3 fed with fucose, line 4-GMDSKO #4, line 5-GMDSKO #4 fed with fucose, line 6-TSTA3KO #10, line 7-TSTA3KO #10 fed with fucose, line 8-TSTA3KO #42, line 9-TSTA3KO #42 fed with fucose. 40 µg of each cell lysate was applied to western blotting with an anti-FPGT antibody.

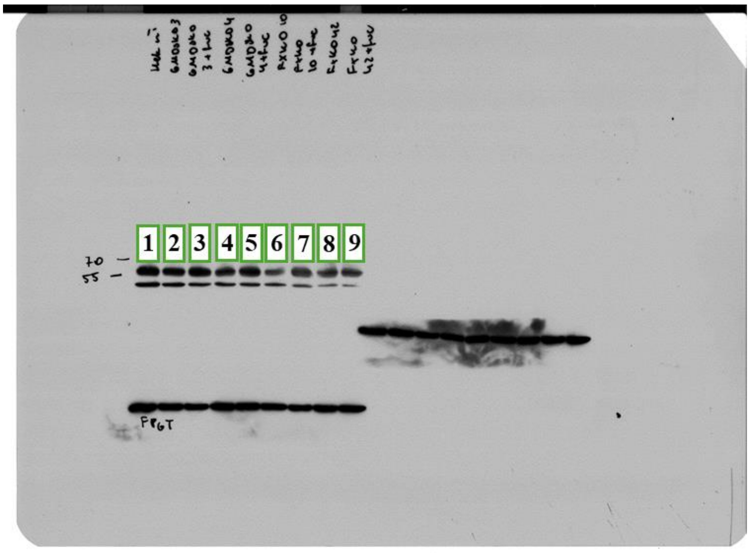

- Used as an insert in figure 4A: western blotting of GAPDH (loading control)

The MW marker is marked on the left side of the line 1 (40 kDa, 35 kDa and 25 kDa). The order of samples from left to right: line 1-HEK293T WT (wild type), line 2-GMDSKO #3, line 3-GMDSKO #3 fed with fucose, line 4-GMDSKO #4, line 5-GMDSKO #4 fed with fucose, line 6-TSTA3KO #10, line 7-TSTA3KO #10 fed with fucose, line 8-TSTA3KO #42, line 9-TSTA3KO #42 fed with fucose. 40 µg of each cell lysate was applied to western blotting with an anti-GAPDH antibody.

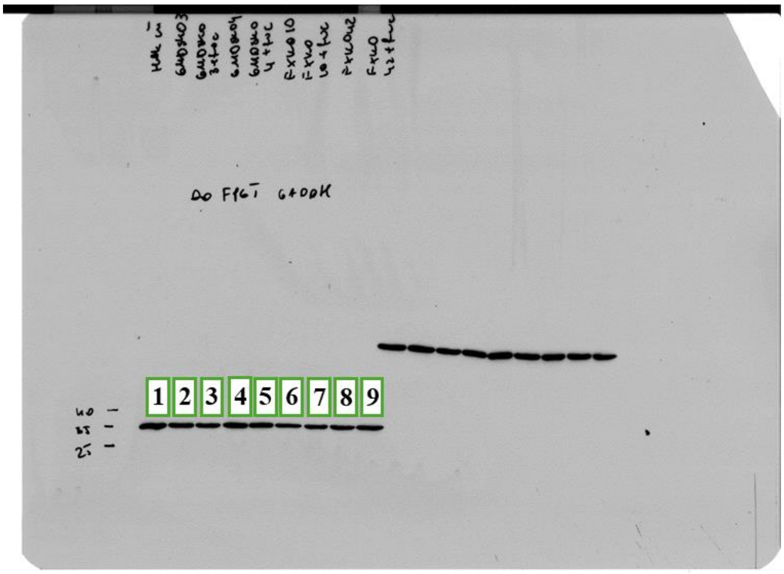

- Used as an insert in figure 4B: western blotting of FCSK

The MW marker is marked on the left side of the line 1 (130 kDa and 100 kDa). The order of samples from left to right: line 1-HEK293T WT (wild type), line 2-GMDSKO #3, line 3 - GMDSKO #3 fed with fucose, line 4-GMDSKO #4, line 5-GMDSKO #4 fed with fucose, line 6-TSTA3KO #10, line 7-TSTA3KO #10 fed with fucose, line 8-TSTA3KO #42, line 9-TSTA3KO #42 fed with fucose. 40 µg of each cell lysate was applied to western blotting with an anti-FCSK antibody.

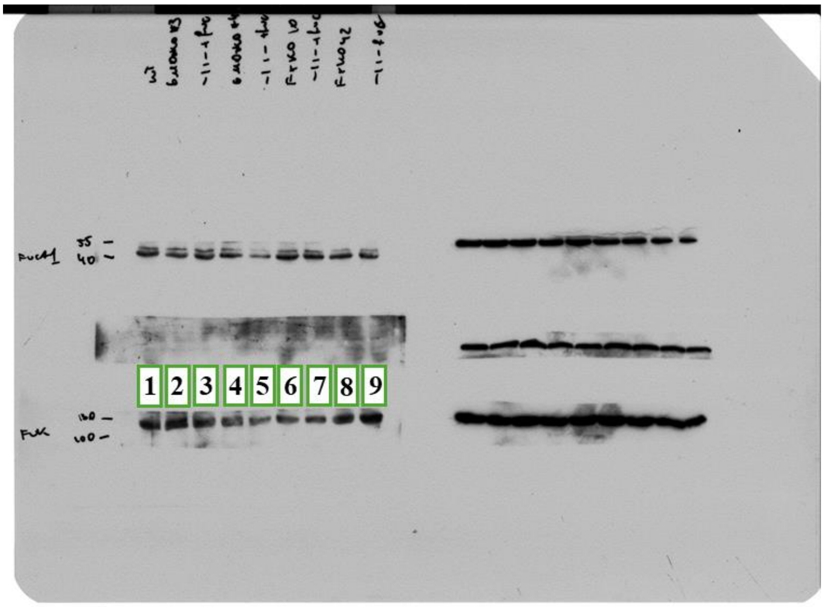

- Used as an insert in figure 4B: western blotting of GAPDH (loading control)

The MW marker is marked on the left side of the line 1 (40 kDa, 35 kDa and 25 kDa). The order of samples from left to right: line 1-HEK293T WT (wild type), line 2-GMDSKO #3, line-3 GMDSKO #3 fed with fucose, line 4-GMDSKO #4, line 5-GMDSKO #4 fed with fucose, line 6-TSTA3KO #10, line 7-TSTA3KO #10 fed with fucose, line 8-TSTA3KO #42, line 9-TSTA3KO #42 fed with fucose. 40 µg of each cell lysate was applied to western blotting with an anti-GAPDH antibody.

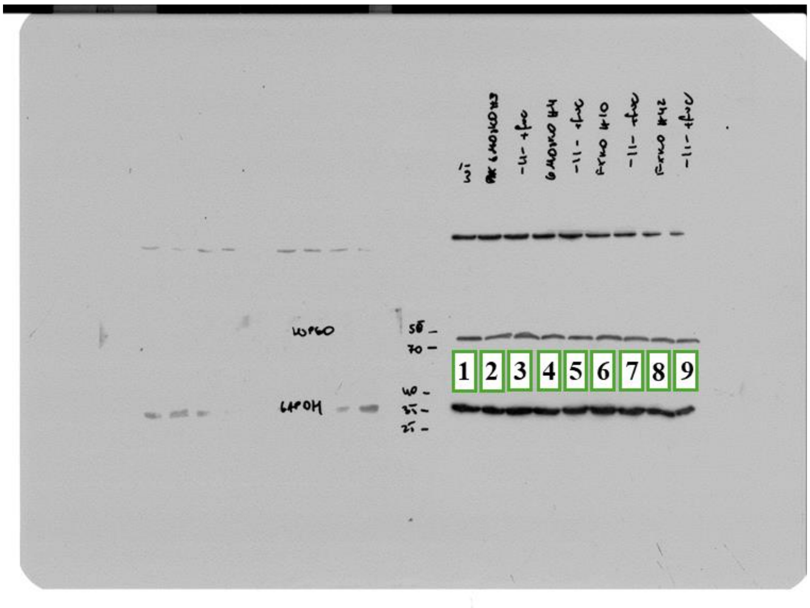

- Used as an insert in figure 4C: western blotting of GMDS

The MW marker is marked on the left side of the line 1 (40 kDa 35 kDa and 25 kDa). The order of samples from left to right: line 1-HEK293T WT (wild type), line 2-FCSKKO #12, line 3-FCSKKO #45. 40 µg of each cell lysate was applied to western blotting with an anti-GMDS antibody.

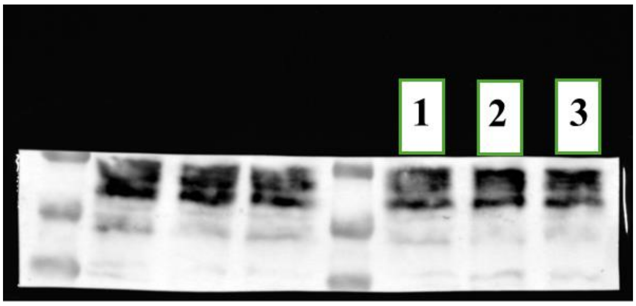

- Used as an insert in figure 4C: western blotting of HSP60 (loading control)

The MW marker is marked on the left side of line 1 (70 kDa and 55 kDa). The order of samples from left to right: line 1-HEK293T WT (wild type), line 2-FCSKKO #12, and line 3- FCSKKO #45. 40 µg of each cell lysate was applied to western blotting with an anti-HSP60 antibody.

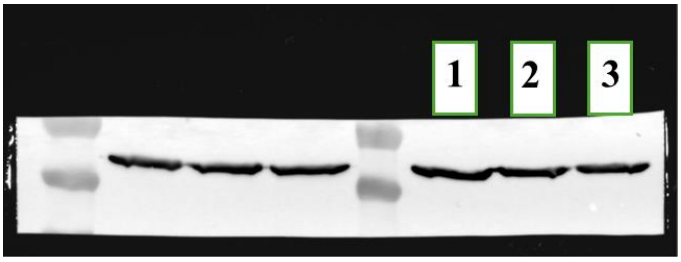

- Used as an insert in figure 4D: western blotting of TSTA3

The MW marker is marked on the left side of the line 1 (40 kDa, 35 kDa and 25 kDa). The order of samples from left to right: line 1-HEK293T WT (wild type), line 2-FCSKKO #12, line 3-FCSKKO #45. 40 µg of each cell lysate was applied to western blotting with an anti-TSTA3 antibody.

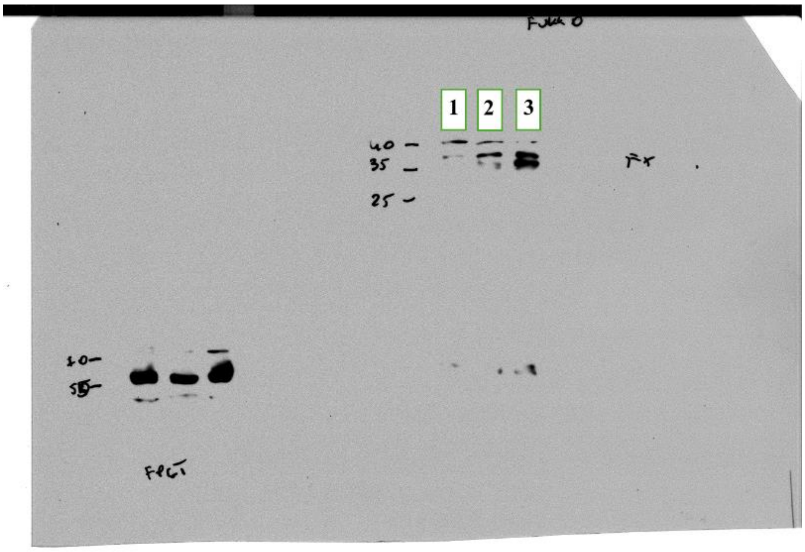

- Used as an insert in figure 4D: western blotting of GAPDH (loading control)

The MW marker is marked on the left side of the line 1 (40 kDa ,35 kDa and 25 kDa). The order of samples from left to right: line 1-HEK293T WT (wild type), line 2-FCSKKO #12, line 3-FCSKKO #45. 40 µg of each cell lysate was applied to western blotting with an anti-GAPDH antibody.

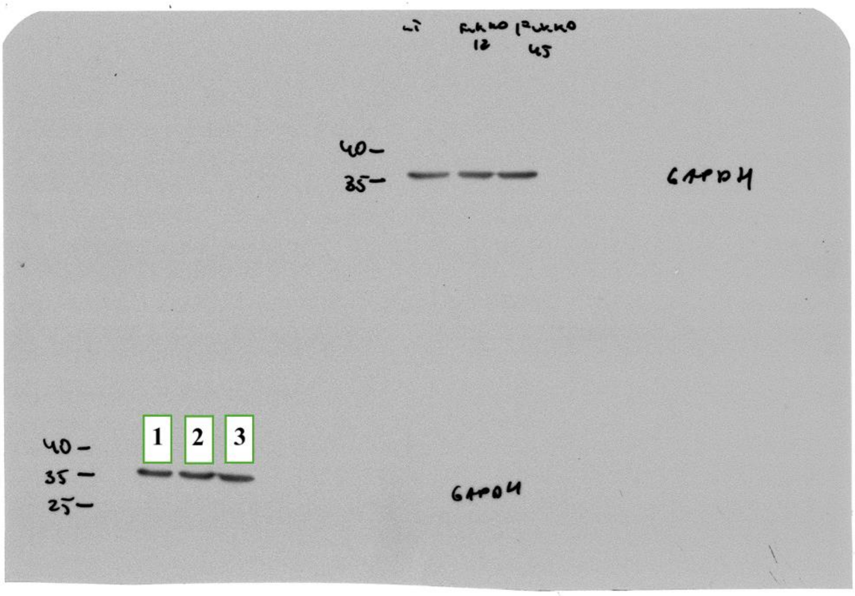

Page 13.

- Used as an insert in figure 5C: western blotting of HA tag

The MW marker is marked on the left side of the line 1 (130 kDa, 100 kDa and 70 kDa). The order of samples from left to right: line 1- GMDSKO, line 2 -GMDSKO + HAFCSK, line 3-TSTA3KO, line 4- TSTA3KO + HAFCSK. 20 µg of each cell lysate was applied to western blotting with an anti-HA antibody.

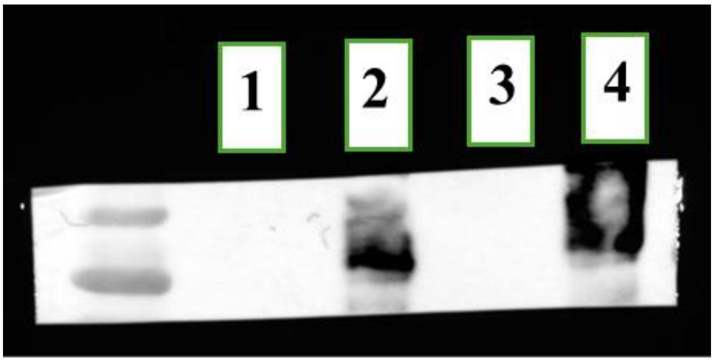

- Used as an insert in figure 5C: western blotting of HSP60 (loading control)

The MW marker is marked on the left side of the line 1 (70 kDa and 50 kDa). The order of samples from left to right: line 1- GMDSKO, line 2 -GMDSKO + HAFCSK, line 3- TSTA3KO, line 4- TSTA3KO + HAFCSK. 20 µg of each cell lysate was applied to western blotting with an anti-HSP60 antibody.

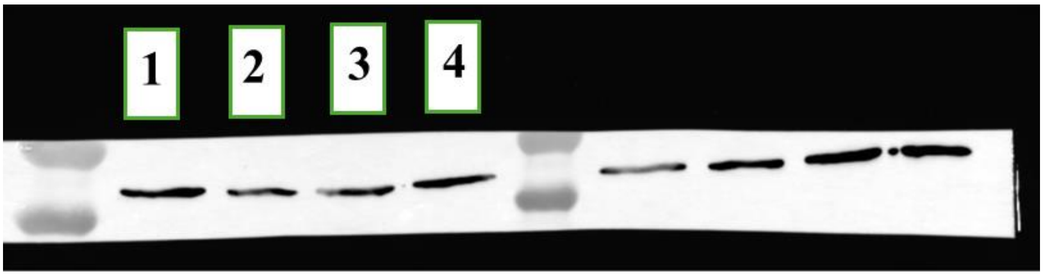

- Used as an insert in figure 5D: western blotting of FCSK (longer exposure)

The MW marker is marked on the left side of the line 1 (130 kDa, 100 kDa and 70 kDa). The order of samples from left to right: line 1- GMDSKO, line 2 -GMDSKO + HAFCSK, line 3- TSTA3KO, line 4- TSTA3KO + HAFCSK. 20 µg of each cell lysate was applied to western blotting with an anti-FCSK antibody.

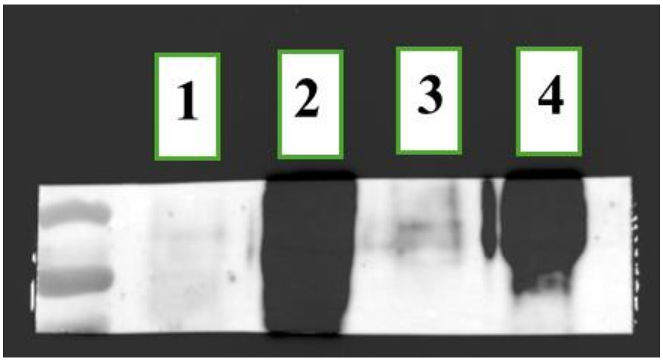

- Used as an insert in figure 5D: western blotting of FCSK (shorter exposure)

The MW marker is marked on the left side of the line 1 (130 kDa, 100 kDa and 70 kDa). The order of samples from left to right: line 1- GMDSKO, line 2 -GMDSKO + HAFCSK, line 3- TSTA3KO, line 4- TSTA3KO + HAFCSK. 20 µg of each cell lysate was applied to western blotting with an anti-FCSK antibody.

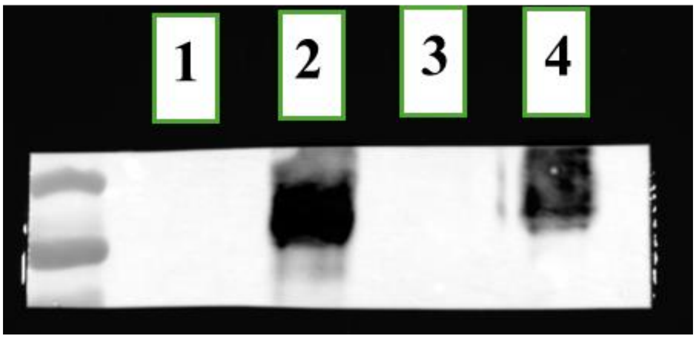

- Used as an insert in figure 5D: western blotting of HSP60 (loading control)

The MW marker is marked on the left side of the line 1 (70 kDa and 50 kDa). The order of samples from left to right: line 1- GMDSKO, line 2 -GMDSKO + HAFCSK, line 3- TSTA3KO, line 4- TSTA3KO + HAFCSK. 20 µg of each cell lysate was applied to western blotting with an anti-HSP60 antibody.

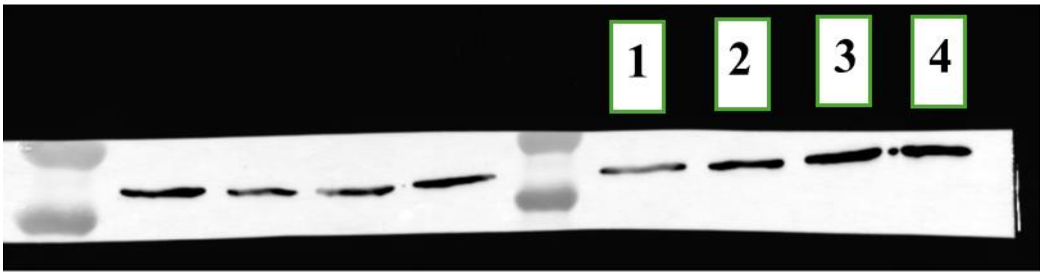

Page 16.

- Used as an insert in figure 6C: western blotting of GLUT1

The MW marker is marked on the left side of the line 1 (55 kDa, 40 kDa and 35 kDa). The order of samples from left to right: line 1-HEK293T WT (wild type), line 2-GMDSKO #3, line 3 -GMDSKO #3 fed with fucose, line 4-GMDSKO #4, line 5-GMDSKO #4 fed with fucose, line 6-TSTA3KO #10, line 7-TSTA3KO #10 fed with fucose, line 8-TSTA3KO #42, line 9-TSTA3KO #42 fed with fucose. 40 µg of each cell lysate was applied to western blotting with an anti-GLUT1 antibody.

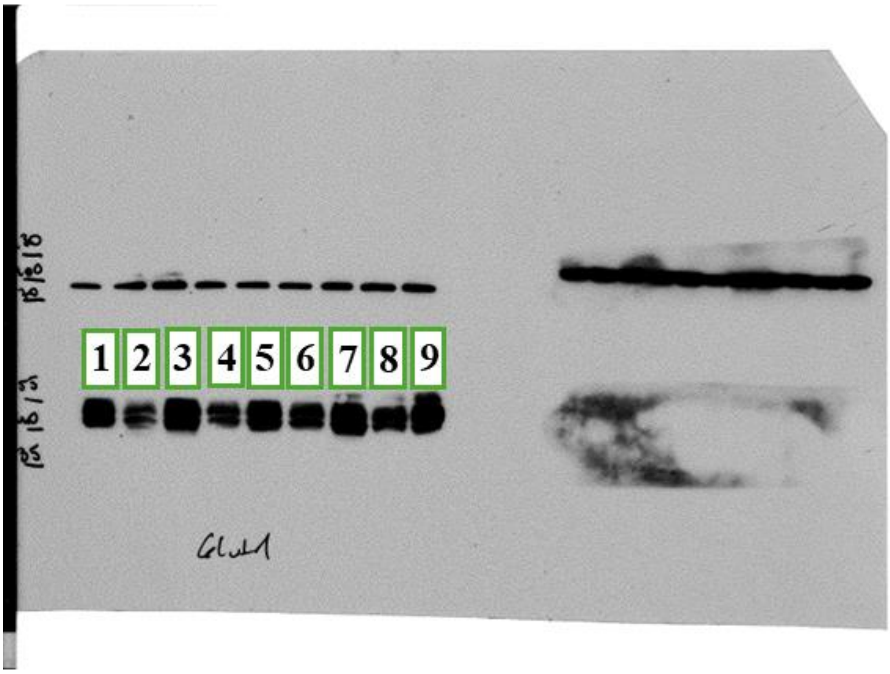

- Used as an insert in figure 6C: western blotting of calnexin (loading control)

The MW marker is marked on the left side of the line 1 (130 kDa, 100 kDa and 70 kDa). The order of samples from left to right: line 1-HEK293T WT (wild type), line 2-GMDSKO #3, line 3 -GMDSKO #3 fed with fucose, line 4-GMDSKO #4, line 5-GMDSKO #4 fed with fucose, line 6-TSTA3KO #10, line 7-TSTA3KO #10 fed with fucose, line 8-TSTA3KO #42, line 9-TSTA3KO #42 fed with fucose. 40 µg of each cell lysate was applied to western blotting with an anti-calnexin antibody.

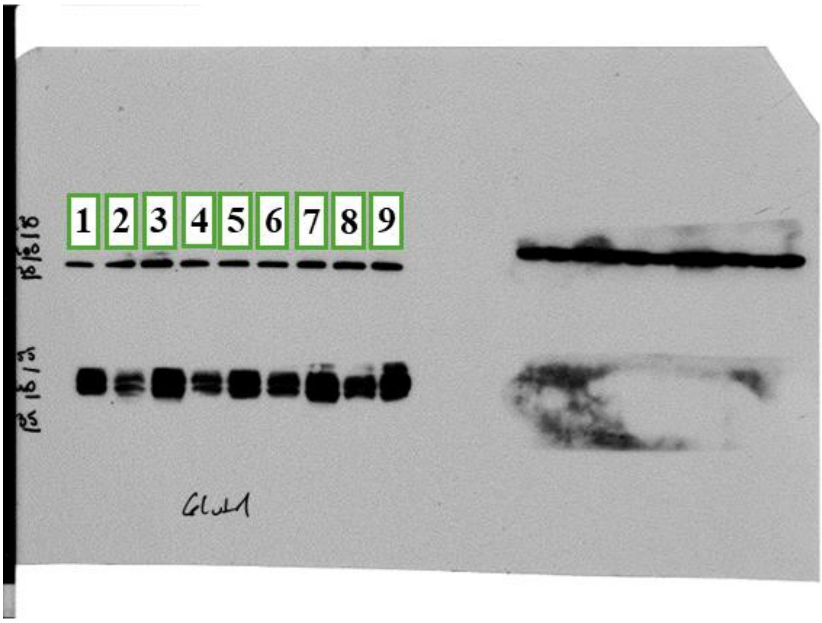

- Used as an insert in figure 6D: western blotting of CaSR

The MW marker is marked on the left side of the line 1 (170 kDa, 130 kDa, 100 kDa and 70 kDa). The order of samples from left to right: line 1-HEK293T WT (wild type), line 2-GMDSKO #3, line 3 -GMDSKO #3 fed with fucose, line 4-GMDSKO #4, line 5-GMDSKO #4 fed with fucose, line 6-TSTA3KO #10, line 7-TSTA3KO #10 fed with fucose, line 8-TSTA3KO #42, line 9-TSTA3KO #42 fed with fucose. 40 µg of each cell lysate was applied to western blotting with an anti-CaSR antibody.

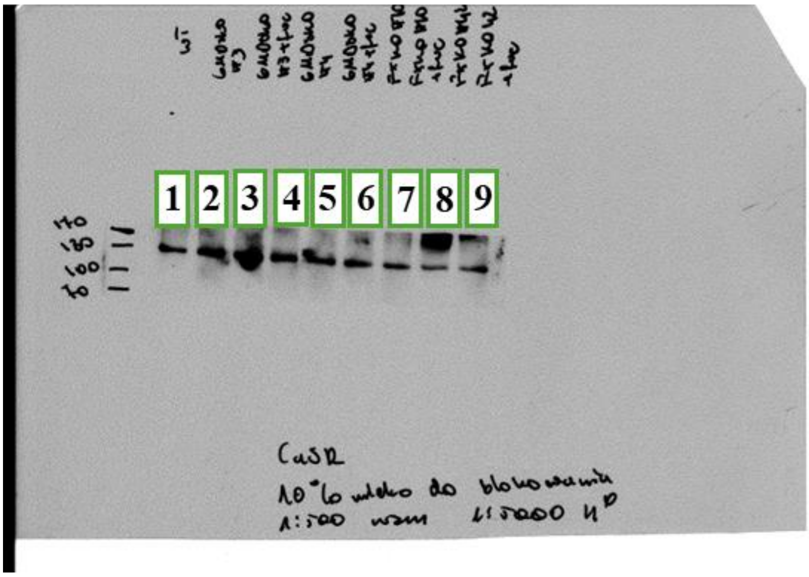

- Used as an insert in figure 6D: western blotting of GAPDH (loading control)

The MW marker is marked on the left side of the line 1 (40 kDa, 35 kDa and 25 kDa). The order of samples from left to right: line 1-HEK293T WT (wild type), line 2-GMDSKO #3, line 3 - GMDSKO #3 fed with fucose, line 4-GMDSKO #4, line 5-GMDSKO #4 fed with fucose, line 6-TSTA3KO #10, line 7-TSTA3KO #10 fed with fucose, line 8-TSTA3KO #42, line 9-TSTA3KO #42 fed with fucose. 40 µg of each cell lysate was applied to western blotting with an anti-GAPDH antibody.

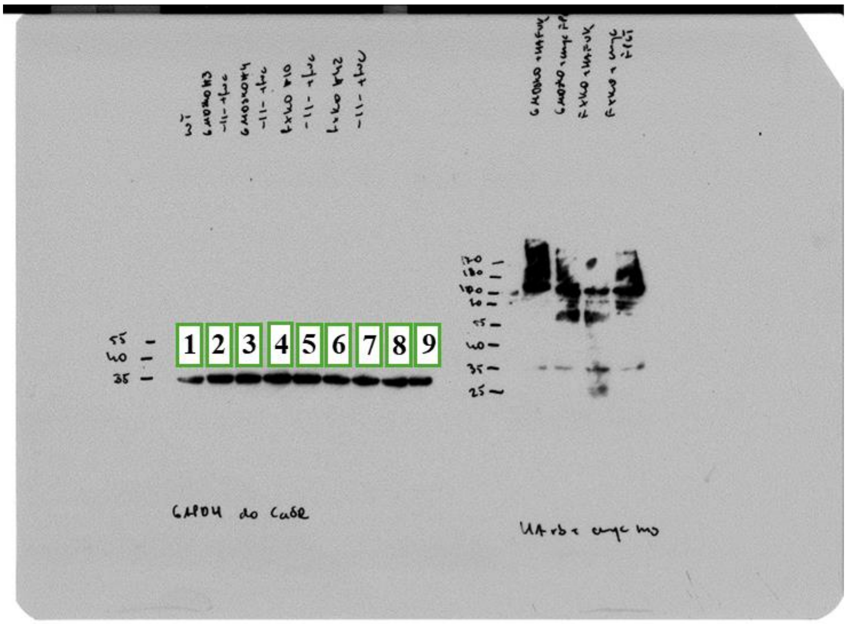

- Used as an insert in figure 6E: western blotting of GLUT1

The MW marker is marked on the left side of the line 1 (40 kDa, 35 kDa and 25 kDa). The order of samples from left to right: line 1-HEK293T WT (wild type), line 2-FCSKKO #12, line 3-FCSKKO #45. 40 µg of each cell lysate was applied to western blotting with an anti-GLUT1 antibody.

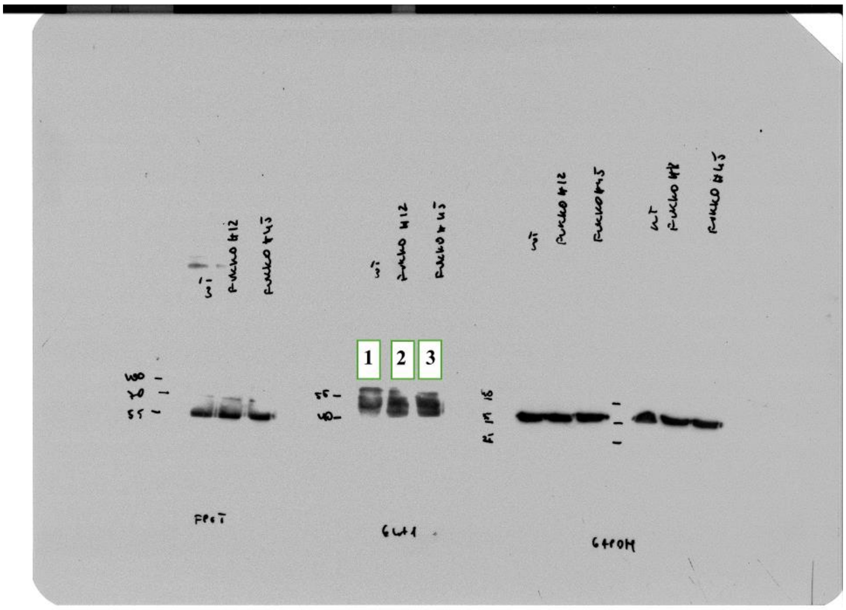

- Used as an insert in figure 6E: western blotting of GAPDH (loading control)

The MW marker is marked on the left side of the line 1 (40 kDa ,35 kDa and 25 kDa). The order of samples from left to right: line 1-HEK293T WT (wild type), line 2-FCSKKO #12, line 3-FCSKKO #45. 40 µg of each cell lysate was applied to western blotting with an anti-GAPDH antibody.

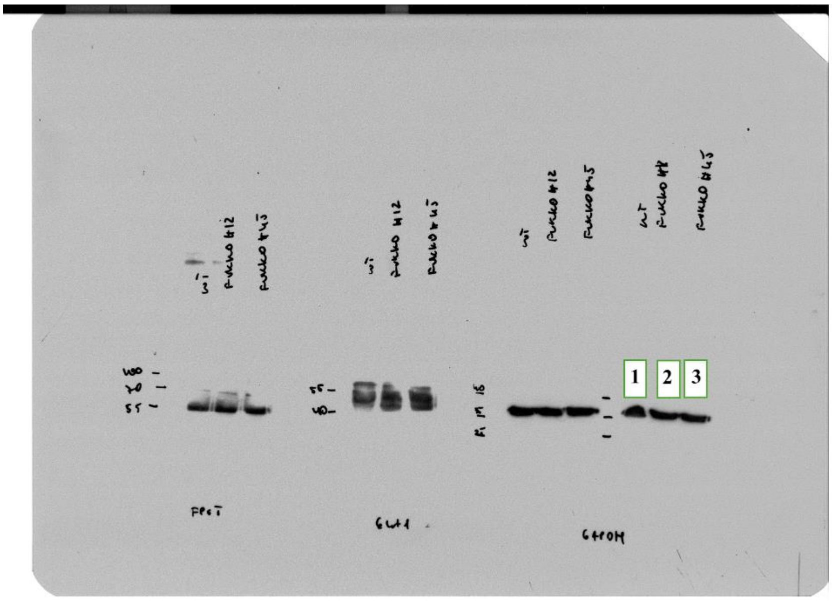

Page 17.

- Used as an insert in figure 7A: western blotting of FUOM

The MW marker is marked on the left side of the line 1 (25 kDa and 15 kDa). The order of samples from left to right: line 1-HEK293T WT (wild type), line 2-GMDSKO #3, line 3 -GMDSKO #3 fed with fucose, line 4-GMDSKO #4, line 5-GMDSKO #4 fed with fucose, line 6-TSTA3KO #10, line 7-TSTA3KO #10 fed with fucose, line 8-TSTA3KO #42, line 9-TSTA3KO #42 fed with fucose. 40 µg of each cell lysate was applied to western blotting with an anti-FUOM antibody.

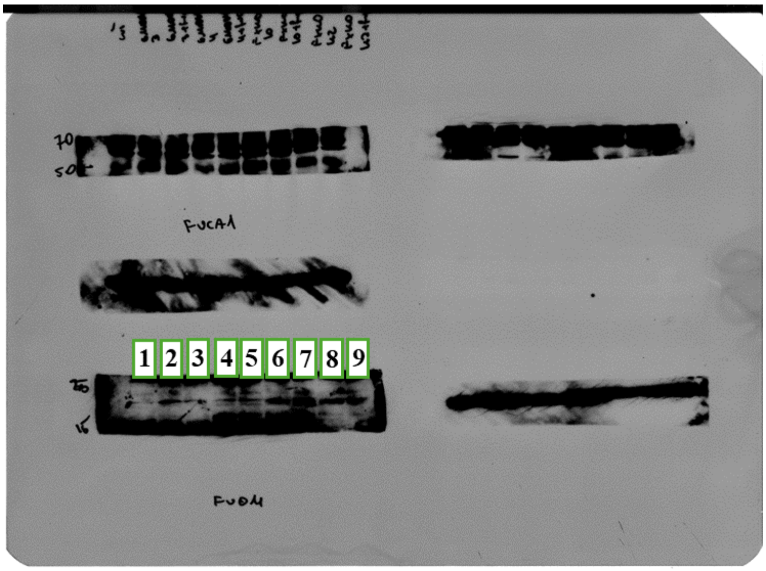

- Used as an insert in figure 7A: western blotting of calnexin (loading control)

The MW marker is marked on the left side of the line 1 (100 kDa and 70 kDa). The order of samples from left to right: line 1-HEK293T WT (wild type), line 2-GMDSKO #3, line 3 - GMDSKO #3 fed with fucose, line 4-GMDSKO #4, line 5-GMDSKO #4 fed with fucose, line 6-TSTA3KO #10, line 7-TSTA3KO #10 fed with fucose, line 8-TSTA3KO #42, line 9-TSTA3KO #42 fed with fucose. 40 µg of each cell lysate was applied to western blotting with an anti-calnexin antibody.

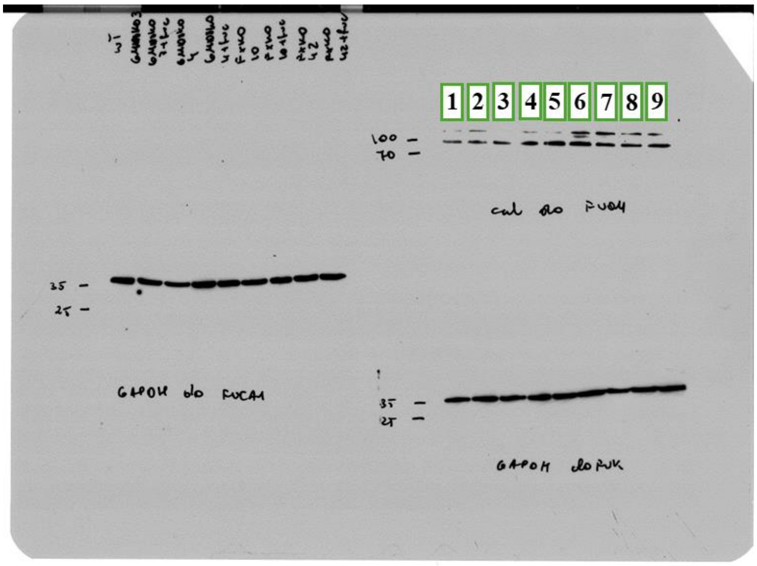

- Used as an insert in figure 7B: western blotting of FUOM

The MW marker is marked on the left side of the line 1 (25 kDa and 15 kDa). The order of samples from left to right: line 1-HEK293T WT (wild type), line 2-FCSKKO #12, line 3-FCSKKO #45. 40 µg of each cell lysate was applied to western blotting with an anti-FUOM antibody.

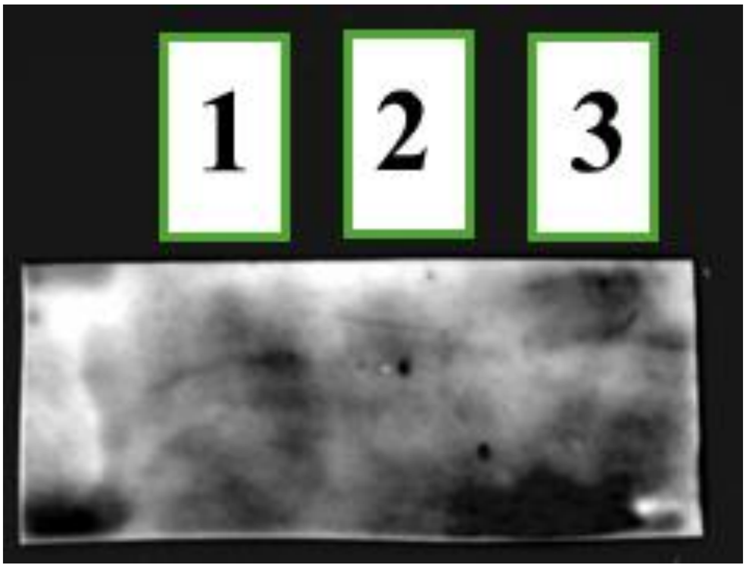

- Used as an insert in figure 7B: western blotting of HSP60 (loading control)

The MW marker is marked on the left side of the line 1 (100 kDa, 70 kDa and 55 kDa). The order of samples from left to right: line 1-HEK293T WT (wild type), line 2-FCSKKO #12, line 3- FCSKKO #45. 40 μg of each cell lysate was applied to western blotting with an anti-HSP60 antibody.

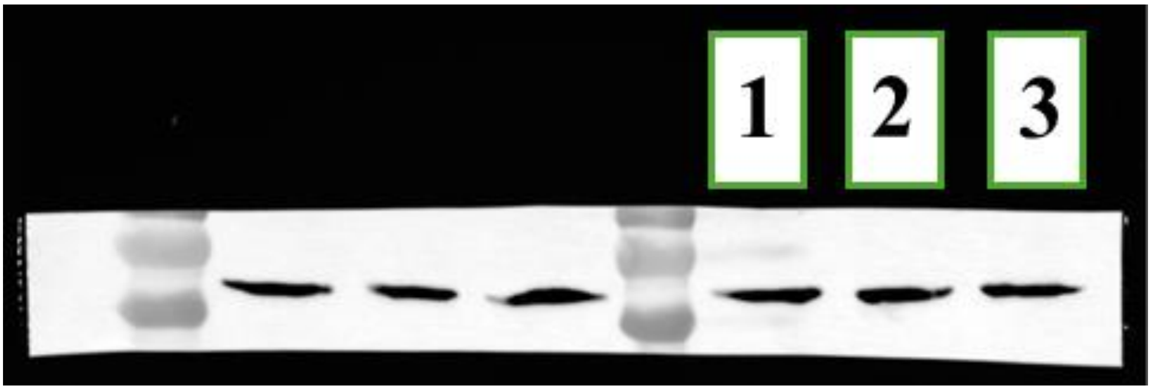

- Used as an insert in figure 7C: western blotting of FUCA1

The MW marker is marked on the left side of the line 1 (55 kDa and 40 kDa). The order of samples from left to right: line 1-HEK293T WT (wild type), line 2-FCSKKO #12, line 3- FCSKKO #45. 40 μg of each cell lysate was applied to western blotting with an anti-FUCA1 antibody.

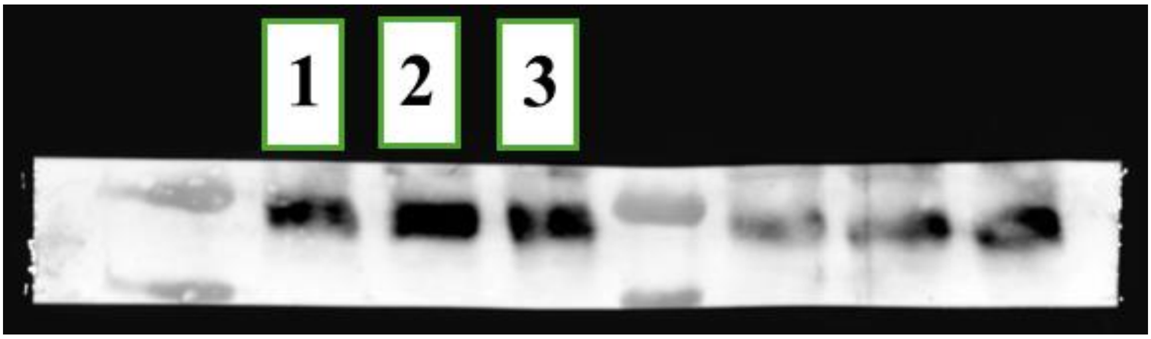

- Used as an insert in figure 7C: western blotting of GAPDH (loading control)

The MW marker is marked on the left side of the line 1 (35 kDa). The order of samples from left to right: line 1-HEK293T WT (wild type), line 2-FCSKKO #12, line 3- FCSKKO #45. 40 μg of each cell lysate was applied to western blotting with an anti-GAPDH antibody.

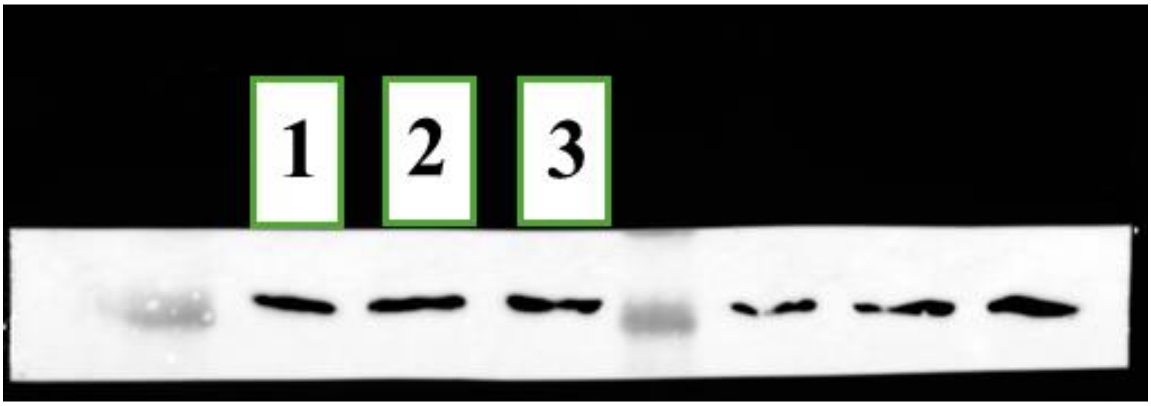

- Used as an insert in figure 7D: western blotting of FUCA1

The MW marker is marked on the left side of the line 1 (55 kDa and 40 kDa). The order of samples from left to right: line 1-HEK293T WT (wild type), line 2-GMDSKO #3, line 3 - GMDSKO #3 fed with fucose, line 4-GMDSKO #4, line 5-GMDSKO #4 fed with fucose, line 6-TSTA3KO #10, line 7-TSTA3KO #10 fed with fucose, line 8-TSTA3KO #42, line 9-TSTA3KO #42 fed with fucose. 40 µg of each cell lysate was applied to western blotting with an anti-FUCA1 antibody.

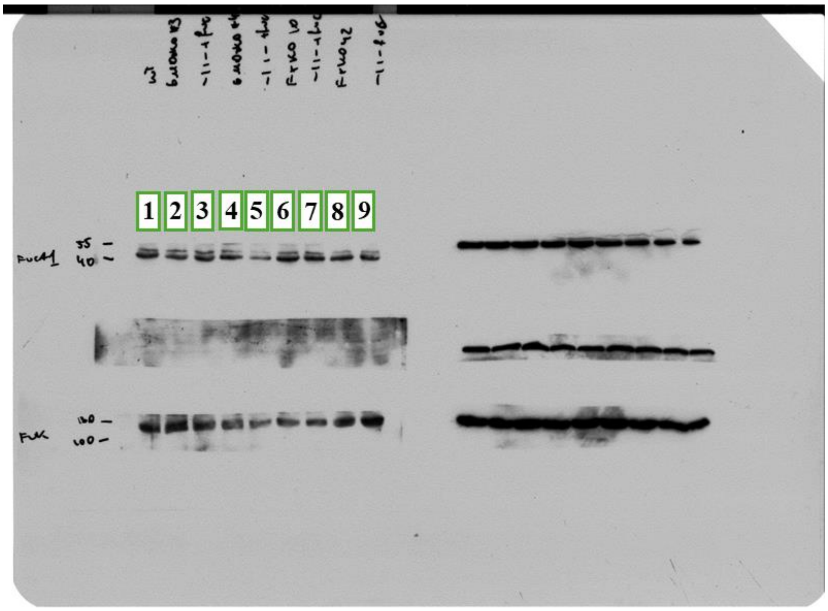

- Used as an insert in figure 7D: western blotting of GAPDH (loading control)

The MW marker is marked on the left side of the line 1 (35 kDa and 25 kDa). The order of samples from left to right: line 1-HEK293T WT (wild type), line 2-GMDSKO #3, line 3 - GMDSKO #3 fed with fucose, line 4-GMDSKO #4, line 5-GMDSKO #4 fed with fucose, line 6-TSTA3KO #10, line 7-TSTA3KO #10 fed with fucose, line 8-TSTA3KO #42, line 9-TSTA3KO #42 fed with fucose. 40 µg of each cell lysate was applied to western blotting with an anti-GAPDH antibody.

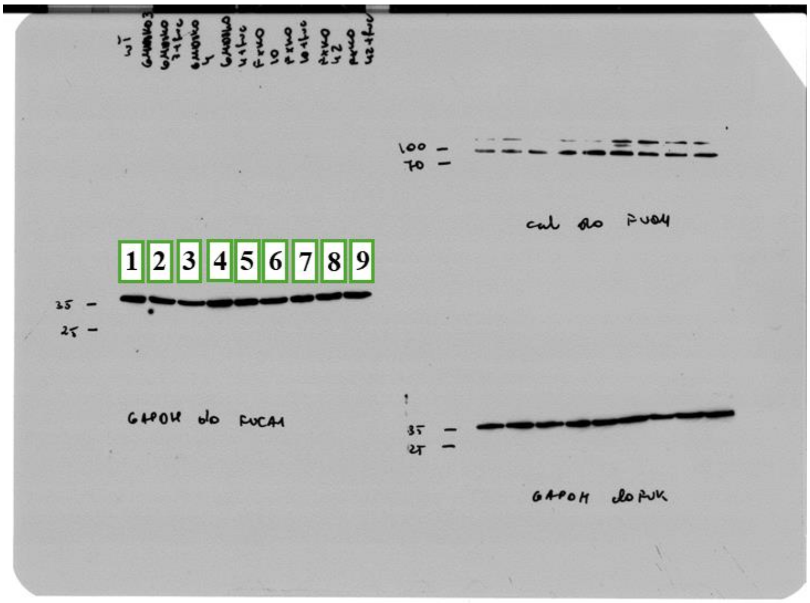

- Used as an insert in figure S1A: western blotting of TSTA3

The MW marker is marked on the left side of the line 1 (40 kDa, 35 kDa and 25 kDa). The order of samples from left to right: line 1-HEK293T WT (wild type), line 2-TSTA3KO #10, line 3-TSTA3KO #42. 20 µg of each cell lysate was applied to western blotting with an anti-TSTA3 antibody.

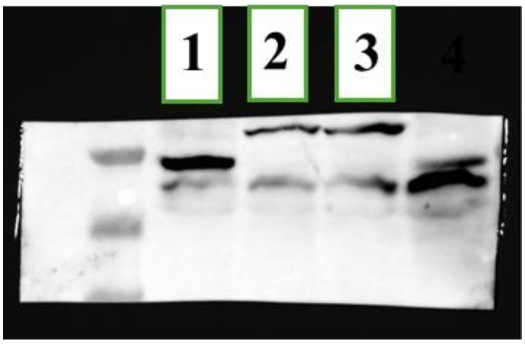

- Used as an insert in figure S1A: western blotting of HSP60 (loading control)

The MW marker is marked on the left side of the line 1 (70 kDa and 55 kDa). The order of samples from left to right: line 1-HEK293T WT (wild type), line 2-TSTA3KO #10, line 3-TSTA3KO #42. 20 µg of each cell lysate was applied to western blotting with an anti-TSTA3 antibody.

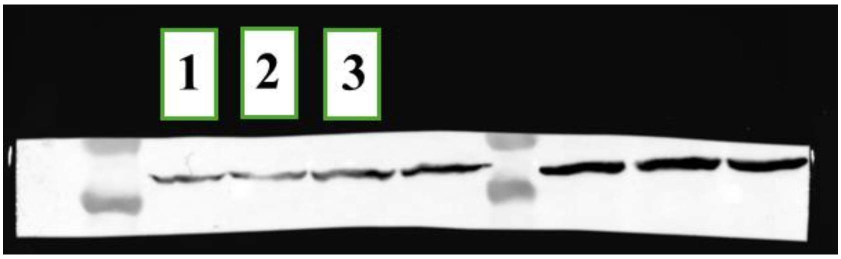

- Used as an insert in figure S1B: western blotting of GMDS (all lines)

The MW marker is marked on the left side of the line 1 (40 kDa, 35 kDa and 25 kDa). The order of samples from left to right: line 1-HEK293T WT (wild type), line 2-GMDSKO #3, line 3-GMDSKO #4. 20 µg of each cell lysate was applied to western blotting with an anti-GMDS antibody.

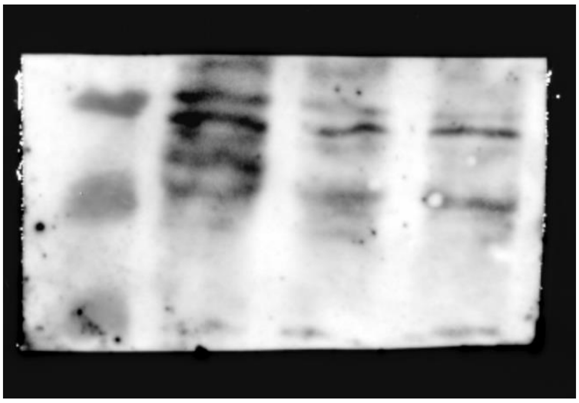

- Used as an insert in figure S1B: western blotting of HSP60 (loading control) (all lines)

The MW marker is marked on the left side of the line 1 (70 kDa and 55 kDa). The order of samples from left to right: line 1-HEK293T WT (wild type), line 2-GMDSKO #3, line 3-GMDSKO #4. 20 µg of each cell lysate was applied to western blotting with an anti-HSP60 antibody.

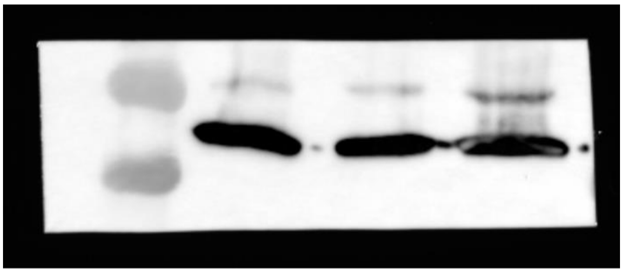

- Used as an insert in figure S1C: western blotting of FCSK

The MW marker is marked on the left side of the line 1 (140 kDa and 95 kDa). The order of samples from left to right: line 1-HEK293T WT (wild type), line 2-FCSKKO #12, line 3-FCSKKO #45. 50 µg of each cell lysate was applied to western blotting with an anti-FCSK antibody.

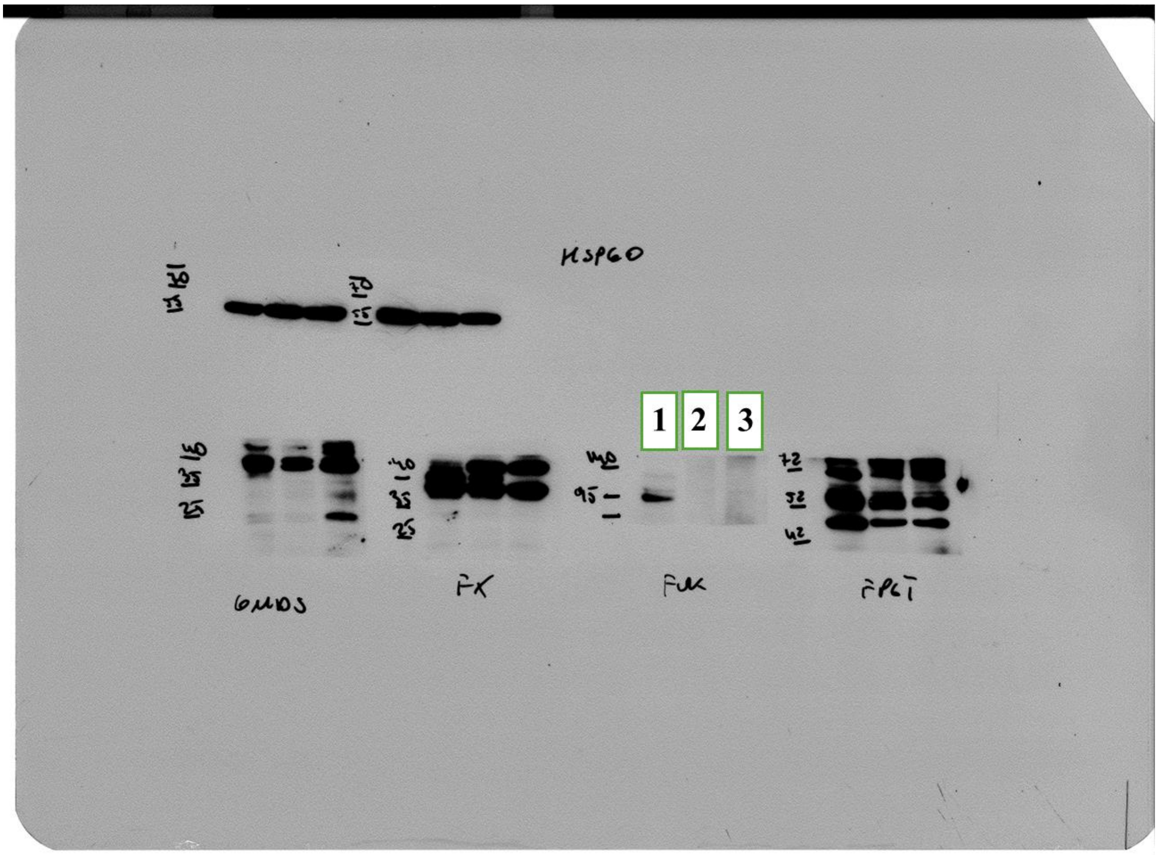

- Used as an insert in figure S1C: western blotting of HSP60 (loading control)

The MW marker is marked on the left side of the line 1 (70 kDa and 55 kDa). The order of samples from left to right: line 1-HEK293T WT (wild type), line 2-FCSKKO #12, line 3-FCSKKO #45. 50 µg of each cell lysate was applied to western blotting with an anti-HSP60 antibody.

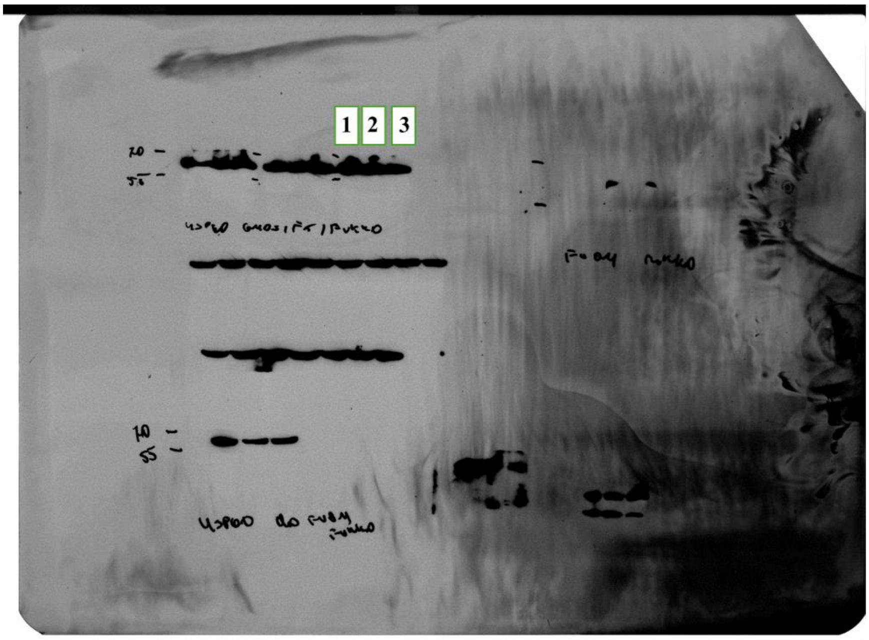

- Used as an insert in figure S1D: electrophoresis of PCR products obtained from One-Step RT-PCR using mRNA

The MW marker is marked on the left side of the line 1 (2000 bp, 1500 bp, 1000 bp, 700 bp, 500 bp, 400 bp, 300 bp, 200 bp, 70 bp). The order of samples from left to right: line 1-HEK293T WT (wild type), line 2-TSTA3KO #10, line 3-TSTA3KO #42. 10 µL of each reaction was applied to electrophoresis.

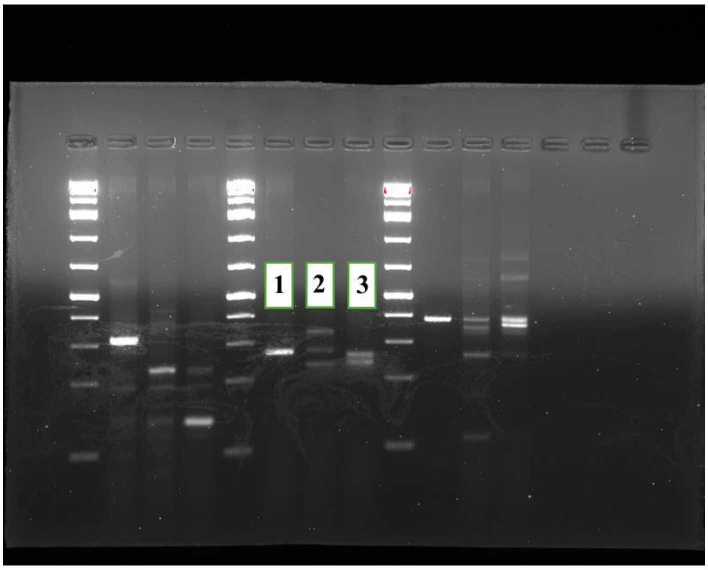

- Used as an insert in figure S1D: electrophoresis of PCR products obtained from PCR reaction using gDNA (all lines)

The MW marker is marked on the left side of the line 1 (2000 bp, 1500 bp, 1000 bp, 700 bp, 500 bp, 400 bp, 300 bp, 200 bp, 70 bp). The order of samples from left to right: line 1-HEK293T WT (wild type), line 2-TSTA3KO #10, line 3-TSTA3KO #42. 10 µL of each reaction was applied to electrophoresis.

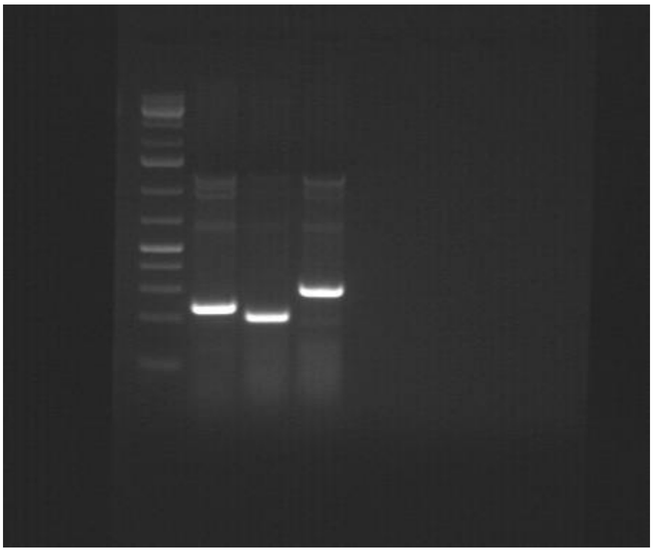

- Used as an insert in figure S1E: electrophoresis of PCR products obtained from One-Step RT-PCR using mRNA

The MW marker is marked on the left side of the line 1 (2000 bp, 1500 bp, 1000 bp, 700 bp, 500 bp, 400 bp, 300 bp, 200 bp, 70 bp). The order of samples from left to right: line 1-HEK293T WT (wild type), line 2-GMDSKO #3, line 3-GMDSKO #4. 10 µL of each reaction was applied to electrophoresis.

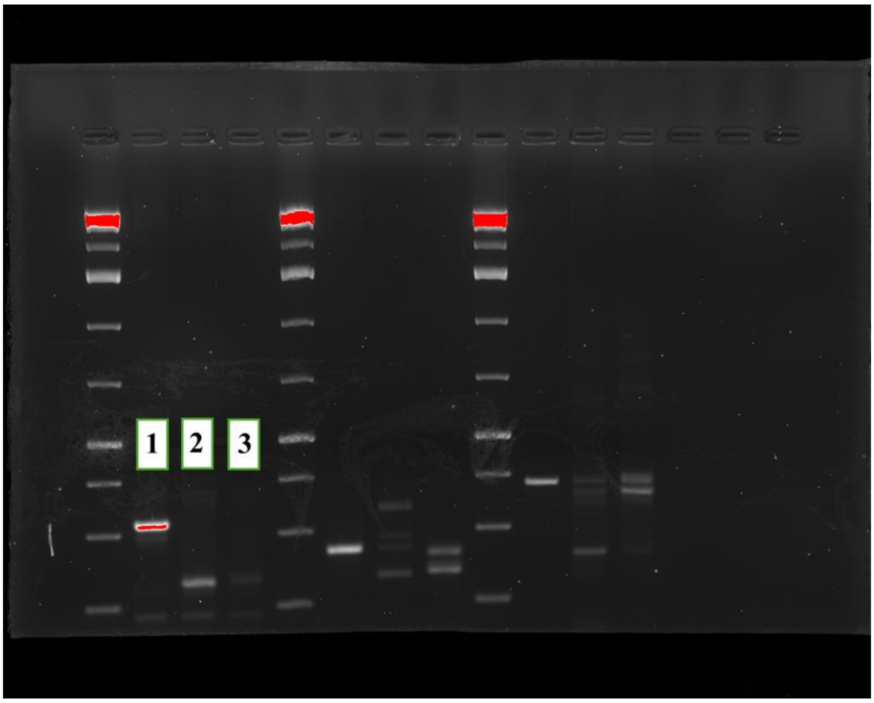

- Used as an insert in figure S1E: electrophoresis of PCR products obtained from PCR reaction using gDNA (all lines)

The MW marker is marked on the left side of the line 1 (2000 bp, 1500 bp, 1000 bp, 700 bp, 500 bp, 400 bp, 300 bp, 200 bp, 70 bp). The order of samples from left to right: line 1-HEK293T WT (wild type), line 2-GMDSKO #3, line 3-GMDSKO #4. 10 µL of each reaction was applied to electrophoresis.

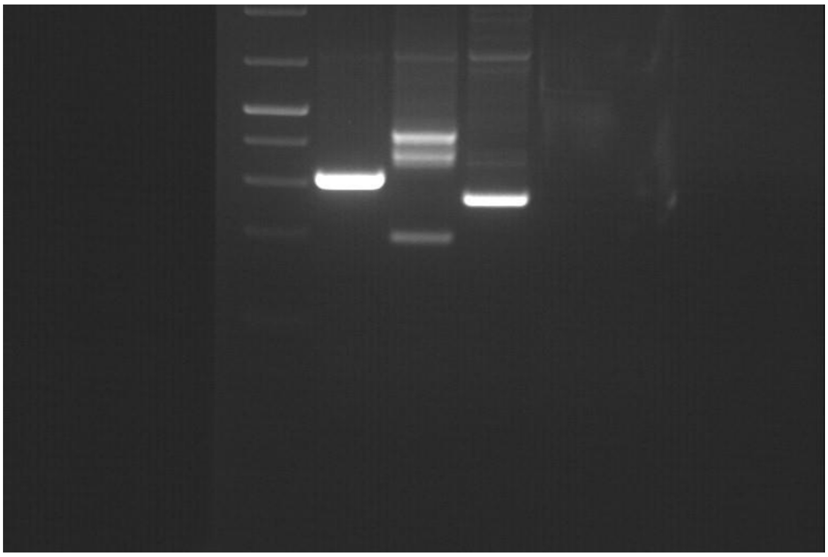

- Used as an insert in figure S1F: electrophoresis of PCR products obtained from One-Step RT-PCR using mRNA

The MW marker is marked on the left side of the line 1 (2000 bp, 1500 bp, 1000 bp, 700 bp, 500 bp, 400 bp, 300 bp, 200 bp, 70 bp). The order of samples from left to right: line 1-HEK293T WT (wild type), line 2-FCSKKO #12, line 3-FCSKKO #45. 10 µL of each reaction was applied to electrophoresis.

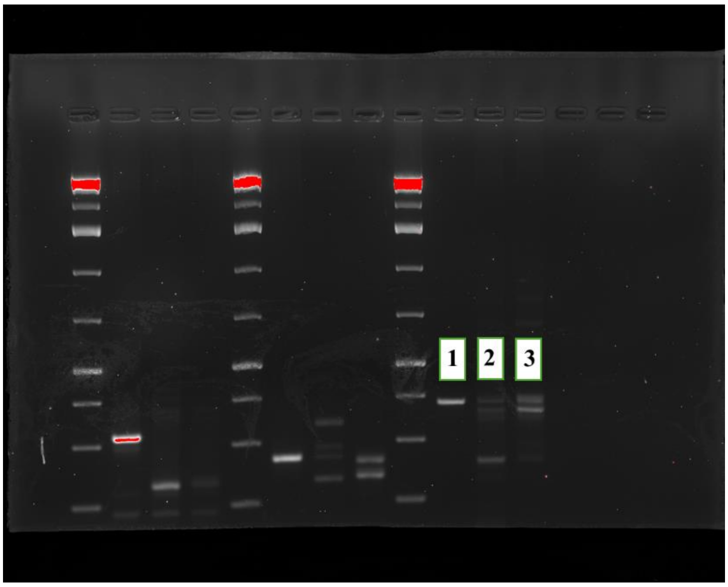

- Used as an insert in figure S1F: electrophoresis of PCR products obtained from PCR reaction using gDNA

The MW marker is marked on the left side of the line 1 (2000 bp, 1500 bp, 1000 bp, 700 bp, 500 bp, 400 bp, 300 bp, 200 bp, 70 bp). The order of samples from left to right: line 1-HEK293T WT (wild type), line 2-FCSKKO #12, line 3-FCSKKO #45. 10 µL of each reaction was applied to electrophoresis.

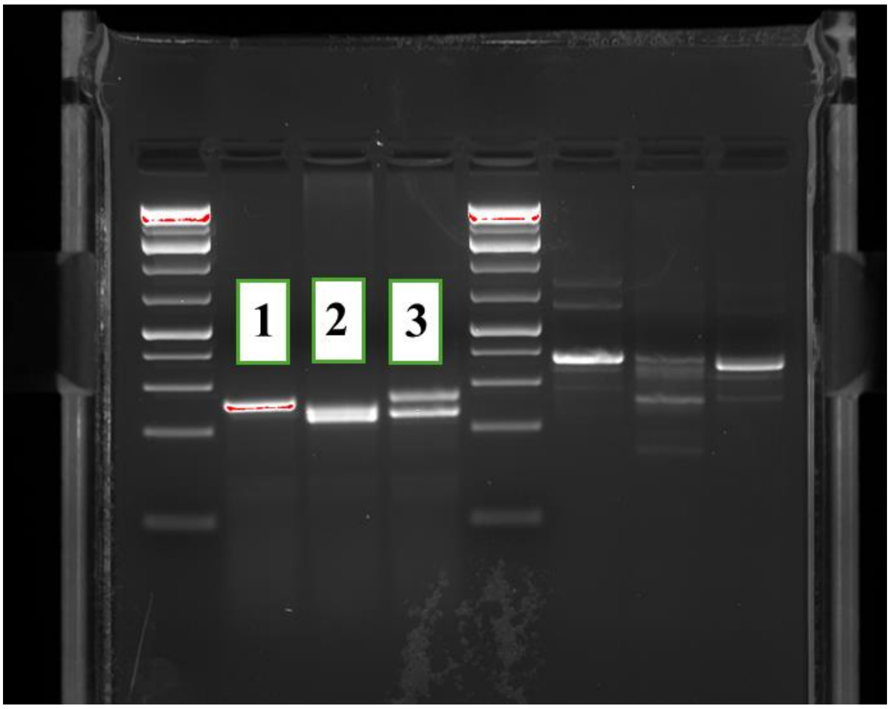

- Used as an insert in figure S3A: western blotting of SLC35C1 (all lines)

The MW marker is marked on the left side of the line 1 (35 kDa and 25 kDa). The order of samples from left to right: line 1-HEK293T WT (wild type), line 2-TSTA3KO #10, line 3-TSTA3KO #10 fed with fucose, line 4-TSTA3KO #42, line 5-TSTA3KO #42 fed with fucose. 40 µg of each cell lysate was applied to western blotting with an anti-SLC35C1 antibody.

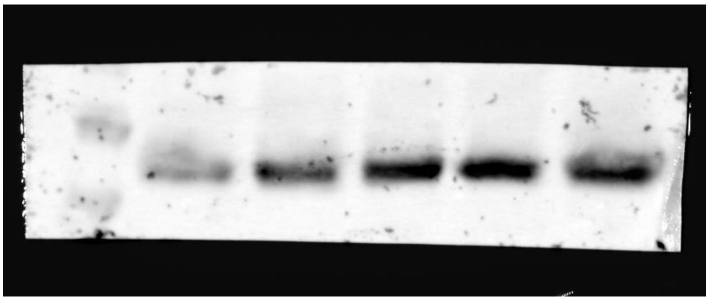

- Used as an insert in figure S3A: Ponceau S staining (all lines)

The MW marker is marked on the left side of the line 1: 260 kDa, 140 kDa, 100 kDa, 70 kDa, 50 kDa, 40 kDa, 35 kDa, and 25 kDa. The order of samples from left to right: line 1-HEK293T WT (wild type), line 2-TSTA3KO #10, line 3-TSTA3KO #10 fed with fucose, line 4-TSTA3KO #42, line 5-TSTA3KO #42 fed with fucose. 40 µg of each cell lysate was applied to Ponceau S staining

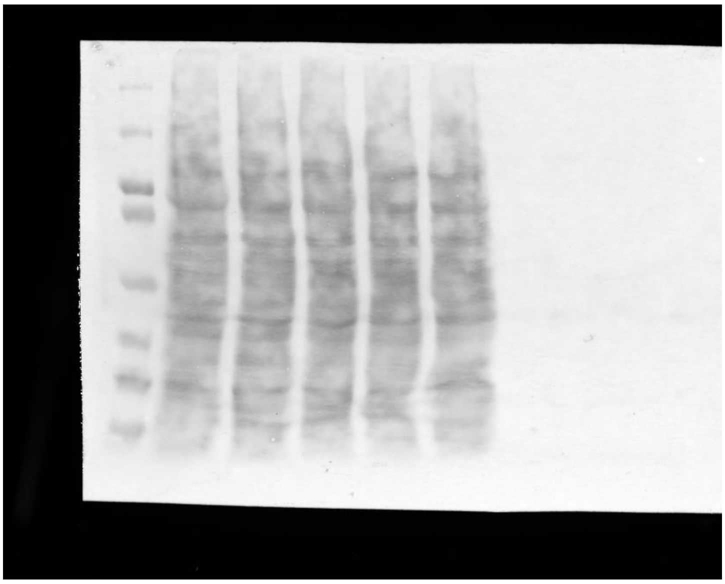

- Used as an insert in figure S3B: western blotting of SLC35C1 (all lines)

The MW marker is marked on the left side of the line 1 (40 kDa, 35 kDa and 25 kDa). The order of samples from left to right: line 1-HEK293T WT (wild type), line 2-GMDSKO #3, line 3-GMDSKO #3 fed with fucose, line 4-GMDSKO #4, line 5-GMDSKO #4 fed with fucose. 40 µg of each cell lysate was applied to western blotting with an anti-SLC35C1 antibody.

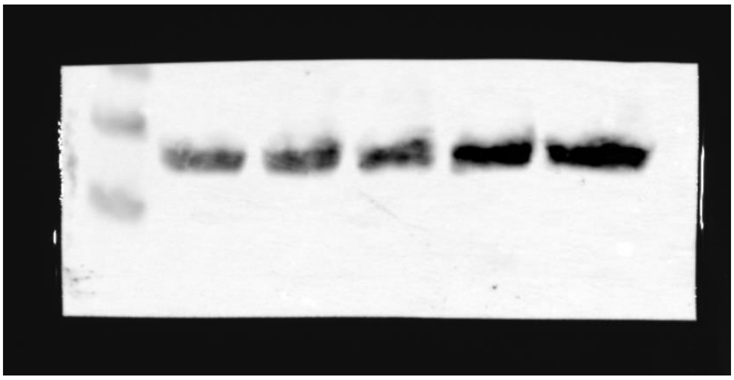

- Used as an insert in figure S3B: Ponceau S staining (all lines)

The MW marker is marked on the left side of the line 1 260 kDa, 140 kDa, 100 kDa, 70 kDa, 50 kDa, 40 kDa, 35 kDa, and 25 kDa. The order of samples from left to right: line 1-HEK293T WT (wild type), line 2-GMDSKO #3, line 3-GMDSKO #3 fed with fucose, line 4-GMDSKO #4, line 5-GMDSKO #4 fed with fucose. 40 µg of each cell lysate was applied to Ponceau S staining.

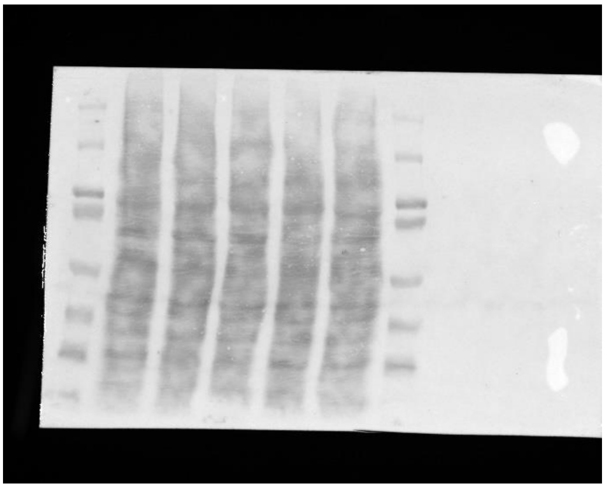

Supplement: S1 File — (ZIP) [file pone.0309450.s004.zip › Supporting Informations_raw_images.pdf]
